# Supplementary material for: Rapid diagnosis and reduced workload for urinary tract infection using flowcytometry combined with direct antibiotic susceptibility testing
Source: PLoS One. 2021 Jul 6;16(7):e0254064. doi: 10.1371/journal.pone.0254064 (PMC8259986; doi:10.1371/journal.pone.0254064)
Supplement: Attachment — Submitted filename: Response to Reviewers .docx [file pone.0254064.s002.docx]

**Rebuttal letter**

We thank the editor and the reviewer for your valuable time and contribution in revising our manuscript. We are grateful for your inputs that will help improve the manuscript. The specific responses to each point raised by the academic editor and reviewer are listed below. We hope that we have satisfyingly addressed them and that the manuscript is now suited for publication.

Sincerely,

On behalf of all authors,

Dr. Hanne M Gilboe, M.D.

**Academic editor:**

1. **Clarification on sample acceptability and algorithm regarding mixed cultures needed.**

Sample acceptability: Fürst Medical Laboratory recommends culture plating and examination of a urine sample in boric acid within 48 hours after voiding, in concordance with European Urinalysis Guideline. Clarification on sample acceptability is included in the manuscript under Method section.

Algorithm regarding mixed cultures: Mixed cultures contain 3 or more strains of bacteria, either Gram positive, Gram negative or both (included in the manuscript).

**Additional Editor comments:**

**Justification and reference for accepting samples collected in boric acid at day 4 should be provided.** Fürst Medical Laboratory recommends culture plating and examination of a urine sample in boric acid within 48 hours after voiding, in concordance with European Urinalysis Guideline. However, as transportation time from rural parts of Norway can be delayed (especially on weekends and holidays) samples are considered for culture plating and examination if it contains information abut clinical infection or/and antibiotics are initiated, as these samples are difficult to reproduce. If the urine sample is culture plated and examined, the clinician will receive a comment that the result is uncertain as the samples was not culture plated within the recommended time frame.

**Please review your reference list to ensure that it is complete and correct. If you have cited papers that have been retracted, please include the rationale for doing so in the manuscript text, or remove these references and replace them with relevant current references. Any changes to the reference list should be mentioned in the rebuttal letter that accompanies your revised manuscript. If you need to cite a retracted article, indicate the article’s retracted status in the References list and also include a citation and full reference for the retraction notice.**

Changes made:

Reference 10: Translation to English and details on place of publication has been added. The link has been removed as only the most recent version of the antibiotic panel is table is available from the Norwegian Working Group on Antibiotics’ web site.

Reference 11: Place of publication has been added.

Reference 12: List of editors, translation from Norwegian to English, place of publication and page numbers have been added.

Reference 13: Date of latest update and date of citation have been added.

Reference 17: English translation has been added.

None of the cited papers have been retraced.

1. **Please ensure that your manuscript meets PLOS ONE's style requirements, including those for file naming.** The manuscript and file naming was edited to comply with the style requirements. We hopefully have no divergences from the style requirements now.
2. **a) We note that you have indicated that data from this study are available upon request. In your revised cover letter, please address the following prompts: If there are ethical or legal restrictions on sharing a de-identified data set, please explain them in detail (e.g., data contain potentially identifying or sensitive patient information) and who has imposed them (e.g., an ethics committee). Please also provide contact information for a data access committee, ethics committee, or other institutional body to which data requests may be sent.** We have found that there are no ethical or legal restrictions on sharing a de-identified data set.

**b) If there are no restrictions, please upload the minimal anonymized data set necessary to replicate your study findings as either Supporting Information files or to a stable, public repository and provide us with the relevant URLs, DOIs, or accession numbers.** We have uploaded the minimal anonymized data set necessary to replicate the study findings as Supporting Information. Thank you for updating the Data Availability statement on our behalf.

3. **We note that you have included the phrase “data not shown” in your manuscript. Unfortunately, this does not meet our data sharing requirements. PLOS does not permit references to inaccessible data. We require that authors provide all relevant data within the paper, Supporting Information files, or in an acceptable, public repository. Please add a citation to support this phrase or upload the data that corresponds with these findings to a stable repository (such as Figshare or Dryad) and provide and URLs, DOIs, or accession numbers that may be used to access these data. Or, if the data are not a core part of the research being presented in your study, we ask that you remove the phrase that refers to these data.** We have removed the phrase as this was not a core part of the research being presented in our study.

Reviewer #1:

Basic Reporting

**Dear Authors
This is a well written study on the use of Sysmex US 5000 in building an algorithm for rapid processing of urine cultures.
The authors have studied 1000 patients with the routine culture and using sysmex 5000 to screen samples for the presence of significant bacterial counts. The study is well designed and the algorithm can help high throughput labs reduce workload and reduce use of plates for urine culture.
A few minor details that may add value to the paper is**

1. **Did the authors look at history of antibiotics in those with sterile pyuria?** No, we did not have this clinical information.

**Could genitourinary tuberculosis be considered in these patients, especially if immunocompromised. I understand the incidence of tuberculosis is very low in Norway, but a clinical suspicion has helped us many a times to identify this problem.** Genitourinary tuberculosis is very rare in Norway. If there is a clinical suspicion that a patient has genitourinary tuberculosis, and the clinician informs the laboratory of this, the sample is forwarded to a laboratory that analyzes for this.

**2. What was the acceptability criteria for urine samples collected in boric acid containers? Usually samples beyond 72 hours are not accepted. Why were samples processed even 4 days after collection.** Fürst Medical Laboratory recommends culture plating and examination of a urine sample in boric acid within 48 hours after voiding, in concordance with European Urinalysis Guideline. However, as transportation time from rural parts of Norway can be delayed (especially on weekends and holidays) samples are considered for culture plating and examination if it contains information abut clinical infection or/and antibiotics are initiated, as these samples are difficult to reproduce. If the urine sample is culture plated and examined, the clinician will receive a comment that the result is uncertain as the samples was not culture plated within the recommended time frame.

**Further, did the authors look at the WBC breakdown in samples that were collected later. Did that impact results especially when analyzing bacterial counts along with WBC counts?** This is a very relevant comment and could have been done. Unfortunately, we did not examine this. However there was only 4 samples that arrived four days after collection so the material was very limited.

3. **The authors clearly state that Gram positive organisms are yeast do not correlate well with culture in the proposed algorithm. Same goes with mixed cultures. If there is some data on clinical symptoms and the reason for the cultures the algorithm may be further improved. Authors may consider the same**. Yes, we did examine if clinical information and indication for the urine sample could be a useful indication to do direct antibiotic susceptibility testing on the samples. However, only 55-65 % of received urine samples contain relevant clinical information, so it is difficult to use clinical information systematically in any algorithm.
